# Inhibition of JNK/STAT3/NF-KB pathway-mediated migration and clonal formation of lung adenocarcinoma A549 cells by daphnetin

**DOI:** 10.1080/19336918.2024.2418049

**Published:** 2024-10-29

**Authors:** Zhe Lv, Yuna Du, Huiqing Zhang, Hui Fang, Yujie Guo, Lifeng Zeng, Yiguo Chen, Dan Li, Rong Li

**Affiliations:** Department of Clinical Laboratory, Jiangxi Provincial People’s Hospital & The first Affiliated Hospital of Nanchang Medical College, Nanchang, China

**Keywords:** Cell migration, daphnetin, lung adenocarcinoma, MAPK, STAT3

## Abstract

Daphnetin, a coumarin derivative isolated from Daphne odorifera, has anti-tumor effects. The MAPK, STAT3, and NF-κB signaling pathways are closely related to the pathogenesis of lung cancer. To investigate the effect of daphnetin on anti-lung adenocarcinoma A549 cells and its mechanism. The anti-tumor effects of daphnetin on the proliferation, clone formation, migration, and invasion of A549 lung adenocarcinoma cells were investigated. The results showed that daphnetin inhibited the proliferation, colony formation, migration, and invasion of A549 cells through the MAPK/STAT3/NF-KB pathway, and mainly inhibited the clonal formation and migration of A549 cells through the JNK pathway. These results provide a new research direction and theoretical basis for the use of daphnetin in the inhibition of lung adenocarcinoma.

## Introduction

Lung cancer is second only to breast cancer in incidence but has the highest mortality rate. Despite great progress in the pathogenesis and treatment of lung adenocarcinoma, it remains one of the most aggressive malignancies. Among the various methods of cancer treatment, traditional Chinese medicine (TCM) and its natural products have outstanding anti-cancer effects because of their unique advantages of high efficiency and low side effects. Daphnetin (Daph), a natural product, is a coumarin derivative isolated from daphne [1]. Dapnetin has a wide range of biological activities, including anti-inflammatory, antioxidant, anti-cancer, neuroprotective, analgesic et al. [2,3]. Daphnetin was found to regulate the mTOR/AMPK/Akt pathway and plays an important role in ROS-induced apoptosis of ovarian cancer cells [4]. Although there are some reports on the anti-tumor effects of daphnetin, there are limited reports on the anti-cancer effects of daphnetin in lung cancer and its related mechanisms.

The development and progression of lung cancer is a multistep process involving complex intracellular signaling pathways and various cellular proteins [5]. The MAPK pathway is thought to be involved in tumorigenesis of cancer cells, including non-small cell lung cancer [6]. Persistent activation of STAT3 [7] and NF-κB has been shown to be important mechanisms of tumorigenesis in lung cancer [8,9]. Wang et al. found that daphnetin promoted apoptosis in lung adenocarcinoma A549 cells and inhibited proliferation by regulating the Akt/NF-κB signaling pathway [10]. However, there are still few reports on the effect of daphnetin on the migration and invasion of lung adenocarcinoma. Therefore, in this study, we investigated the role of the MAPK/STAT3/NF-κB signaling pathway in the anti-tumor effects of daphnetin and its mechanism in lung adenocarcinoma A549 cells, and clarified the anti-tumor effect of daphnetin and its related mechanism in lung adenocarcinoma A549 cells.

## Materials and methods

### Cell culture and reagents

Human lung adenocarcinoma A549 cells were purchased from Shanghai Fuheng Biotechnology Co., Ltd. The cells were cultured in RIPM-1640 medium (Solarbio) containing penicillin and streptomycin supplemented with 10% (fet bovine serum FBS; Gibco). Cells were cultured in an incubator at 37°C and 5% CO_2_. Daphnetin with purity ≥98%, molecular weight 178.14 Da. The JNK inhibitor SP600125 was purchased from MCE Company. CCK8 Cell Viability Assay Kit was purchased from Yeasen Biotechnology Co., Ltd. Matrix glue was supplied by Corning, Inc. (Corning, NY, USA). Antibodies against ERK1/2 (1:1000), phospho-ERK1/2 (1:1000), GAPDH (1:5000) were purchased from Proteintech. Antibodies against JNK (1:2000), phospho-JNK (1:2000), p38 (1:2000), phospho-p38 (1:2000), STAT3 (1:2000), phospho-STAT3 (1:2000), NF-κB p65 (1:1000), and phospho-NF-κB p65 (1:500) were purchased from Cell Signaling Technology. Antibodies against IκB-α (1:1000), and phospho-IκB (1:1000) were purchased from Abmart Shanghai Co., Ltd.

### Cell treatment

Cells (4 × 10^5^ cells/well) were seeded in 6-well plates and cultured for 24 h until they reached approximately 80% confluence. After the cells were treated with DMSO and different concentrations of daphnetin (0, 5, 10 µg/mL), respectively, 6-well plates were placed in a cell incubator containing 5% CO2 at 37°C for 24 h, and the cells were collected 24 h later. To evaluate the inhibitory effect of the JNK inhibitor, cells were treated with DMSO and different concentrations of JNK inhibitors (0, 2.5, 5, 7.5, 10 µg/mL) for 24 h. To evaluate the effect of the JNK inhibitor and daphnetin, a JNK inhibitor was added to the cells for 1 h, and then DMSO and different concentrations of daphnetin (0, 10 µg/mL) were added to the cells for 24 h. After the cells were treated, 6-well plates were placed in a cell incubator containing 5% CO2 at 37°C, and cells were collected 24 h later.

### Cell viability assay

Cell viability was determined using the CCK8 Cell Viability Assay Kit according to the manufacturer’s instructions of the assay kit. A549 cells were seeded at a density of 5000 cells/well in a 96-well plate overnight, and treated with daphnetin (0, 5, 10, 20, 40, 60 µg/mL) for 24 h. 10 µL CCK8 was added to each well and the cells were cultured at 37°C for 1 h. Absorbance was measured at 450 nm using a microplate reader (Bio-Tek, Vermont).

### Transwell assay

Matrigel (100 µL) was added to the transwell chambers (8 µm) and incubated at 37°C for 6 h. A549 cells (5 × 10^4^ cells) in 200 μL of RPMI-1640 medium without FBS were seeded in the upper part of each chamber on the insert, and 600 μL of RPMI-1640 medium with 10% FBS was placed in the lower chamber. The control group was untreated, DMSO was added to the solvent control group, 10 µg/mL daphnetin was added to the experimental group, and JNK inhibitor and daphnetin were added to the inhibitor group. DMSO, daphnetin, and JNK inhibitor were added to the upper chamber, and the 24-well plate was placed in an incubator for 24 h, liquid in the upper chamber was discarded, and the cells invading the lower chamber were collected.

The membranes were fixed with methanol for 20 min, moistened twice with PBS, stained with 0.1% crystal violet solution for 15 min, moistened again with PBS, and gently wiped off the cells in the upper chamber with a cotton swab. The migrated cells in five randomly chosen fields were imaged and counted under an optical microscope.

### Wound healing assay

A549 cells were seeded into a six‑well plate at a density of 6×10^5^ cells/well. When the cell confluence reached more than 90%, a sterile 200 µL pipette tip was used to create wounds vertically in the wells, and the floating cells were washed away with PBS. The cells were then cultured in RPMI-1640 medium supplemented with 2% fetal bovine serum (FBS). Dimethyl sulfoxide (DMSO), daphnetin, and JNK inhibitors were added to the medium. The morphological changes of the cells at the same position of the wound were observed and captured using a microscope at 0, 24, 48, and 72 h after wound creation to compare the healing speed of each group.

### Clonal formation experiment

A549 cells were seeded at a density of 800 cells/well in a six‑well plate. The next day, the A549 cells were observed to be fully adherent to the cell wall. Dimethyl sulfoxide (DMSO), daphnetin, and JNK inhibitors were added to the medium. The fluid was changed every 4 d, the cell mass (counting ≥50 cells) was observed under the microscope, the cell mass was visible to the naked eye on the cell culture dish, and cell cloning was performed. Cells in each well were fixed with 4% paraformaldehyde for 20 min, washed three times with PBS, stained with 0.1% crystal violet solution for 15 min, washed again with PBS three times, dried, and photographed.

### Western blot assay

Cells were collected, and lysed using RIPA lysis buffer containing PMSF (100:1) to extract proteins. Protein concentration was determined according to the instructions of the BCA Protein Assay Kit. The proteins were separated by 10% SDS-PAGE and electro-transferred to polyvinylidene fluoride (PVDF) membranes. Membranes were blocked with 5% fat-free milk and washed with TBST. After washing the membranes three times, they were placed in a refrigerator at 4°C and incubated with primary antibodies overnight. After washing the membranes three times, they were incubated with secondary antibodies for 2 h at room temperature. An ECL system (Sage Creation) was used to detect the bands.

### Statistical analysis

Statistical software GraphPad Prism 9.0 was used to analyze and plot the experimental data. All data are presented as mean± standard deviation (SD). One-way analysis of variance (ANOVA) was used to compare differences between groups. Results with a *p*- value of <0.05 were considered to indicate statistical significance.

## Results

### Daphnetin inhibits the activation of MAPK signaling pathway

**Figure 1.**
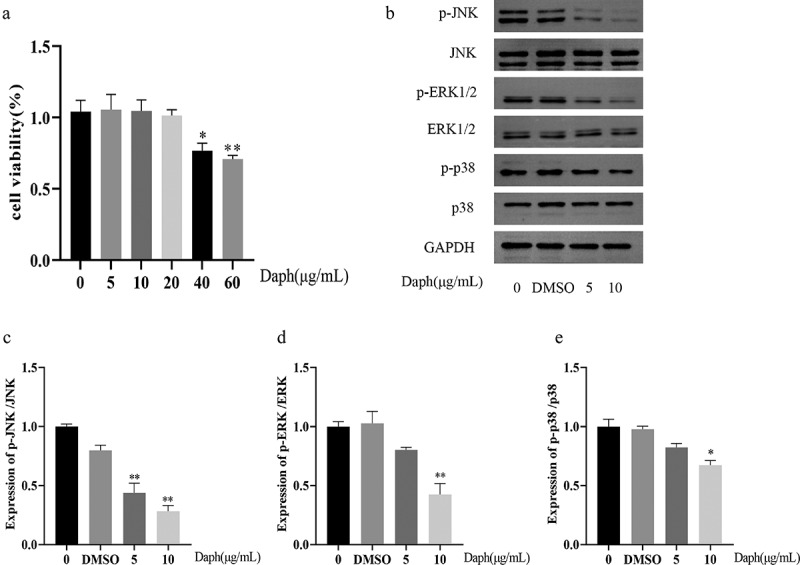
Effect of daphnetin on the MAPK signaling pathway in A549 cells.

To explore the potential toxicity of daphnetin, the viability of A549 cells treated with different concentrations of daphnetin for 24 h was detected using CCK8. The results showed that daphnetin had no significant effect on the viability of A549 cells within a concentration range of 20 µg/mL (Figure 1a). The MAPK signaling pathway is not only involved in stress and inflammatory activation but is also associated with cancer [11]. To explore whether daphnetin play an anti-tumor role by regulating the MAPK signaling pathway, the expression of MAPK signaling pathway-related proteins in A549 cells treated with daphnetin for 24 h was detected by western blotting. The results showed that the phosphorylations of JNK, ERK1/2, and p38 were markedly suppressed by daphnetin in comparison to DMSO group. Compared with the control group, the protein expressions of JNK, ERK1/2, p38, p-JNK, p-ERK1/2 and p-p38 in DMSO group were not significantly different (Figure 1b-e). These results indicated that daphnetin regulates the MAPK signaling pathway by inhibiting the phosphorylation of JNK, ERK1/2, and p38.

(a) The A549 cells were treated with different concentrations of daphnetin for 24 h, and the effect of daphnetin on the activity of A549 cells was detected by CCK8 assay. * indicates the significant difference compared with Control group of 0μg/mL daphnetin (*n* = 3, *p < 0.05,**p < 0.01) (b-e) The A549 cells were treated with DMSO and daphnetin at different concentrations for 24 h, and the cell proteins of control group (0g/mL daphnetin), DMSO solvent control group and experimental group (5,10g/mL daphnetin) were extracted, respectively. The MAPK signaling pathway-related molecules p-JNK, JNK, p-ERK1/2, ERK1/2, p-p38, p38 were detected by Western Blot. * indicates the significant difference compared with DMSO group (*p < 0.05, **p < 0.01)

### Daphnetin and JNK inhibitor inhibit the activation of STAT3 signaling pathway

**Figure 2.**
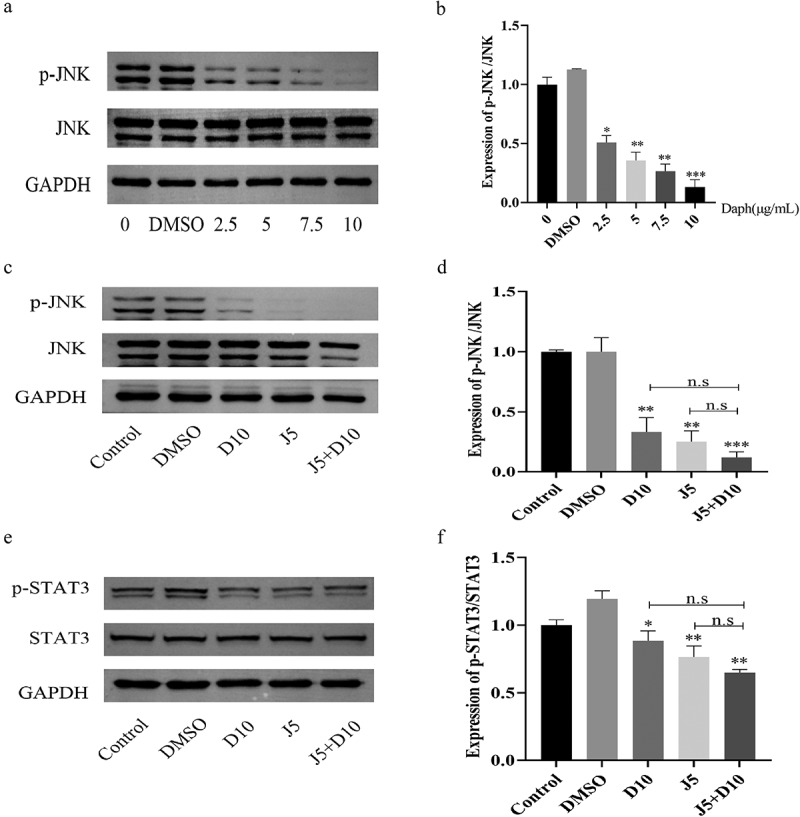
Effects of daphnetin and JNK inhibitor on STAT3 pathway in A549 cells.

According to some studies, individuals with non-small cell lung cancer (NSCLC) show STAT3 activation in their samples or cell lines, which is associated with poor prognosis [7]. The potential relationship between MAPK and STAT3 pathways was further explored using the JNK inhibitor. The results of this study showed that the expression of p-JNK was significantly reduced in JNK inhibitor group compared to DMSO group (Figure 2a,b). Therefore, a concentration of 5 μmol/L JNK inhibitor was selected as the subsequent concentration. The results of this study showed that the expression of p-JNK was significantly reduced in daphnetin group compared to DMSO group (Figure 2c,d). The JNK inhibitor group further reduced the protein expression of p-JNK, and the J5+D10 group (5 μmol/L JNK inhibitor +10 g/mL daphnetin) also showed significantly decreasing tendency (Figure 2c,d). The results of this study showed that the expression of p-STAT3 was significantly reduced in daphnetin group compared to DMSO group (Figure 2e,f). The JNK inhibitor group further reduced the protein expression of p-STAT3, and the J5+D10 group (5 μmol/L JNK inhibitor +10 g/mL daphnetin) also showed significantly decreasing tendency (Figure 2e,f). Taken together, these results indicate that daphnetin and JNK inhibitor could inhibit the phosphorylation of STAT3 and participate in the regulation of the STAT3 signaling pathway, and JNK inhibitor could enhance the inhibitory effect of daphnetin.

A549 cells were treated with DMSO and different concentrations of JNK inhibitors (0, 2.5, 5, 7.5, 10µg/mL) for 24 h. (a-b) The protein expressions of p-JNK and JNK were detected by Western Blot. * indicates the significant difference compared with DMSO group (*p < 0.05, **p < 0.01). A549 cells were pretreated with JNK inhibitor for 1 h, then treated with DMSO and daphnetin with different concentrations for 24 h, and then the cell proteins of Control group (0g/mL daphnetin), DMSO solvent control group and experimental group (10g/mL daphnetin, 5μmol/L JNK inhibitor, 5μmol/L JNK inhibitor+10g/mL daphnetin) were extracted, respectively. (c-d) The protein expressions of p-JNK and JNK were detected by Western Blot. (e-f) The protein expressions of p-STAT3 and STAT3 were detected by Western Blot.* indicates the significant difference compared with DMSO group, and n.s indicates that there is no significant difference between the two groups (*p < 0.05, **p < 0.01).

### Daphnetin and JNK inhibitor inhibit the activation of MAPK signaling pathway

**Figure 3.**
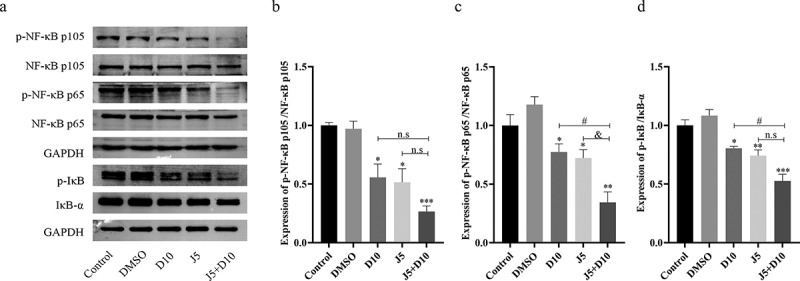
Effects of daphnetin and JNK inhibitor on nf-κB pathway in A549 cells.

NF-κB has a bidirectional effect in non-small cell lung cancer; on the one hand, it plays a crucial role in the immune response, and on the other hand, it promotes inflammation, which triggers the development of tumors [12]. To explore whether daphnetin plays a role in the regulation of the NF-κB signaling pathway, this study further explored the relationship between the MAPK and NF-κB pathways. Changes in the NF-κB pathway were detected using western blotting after protein extraction from A549 cells. The results revealed that the expressions of NF-κB p105, NF-κB p65, and IκB phosphorylation were all markedly suppressed by daphnetin compared to that in the DMSO group. The JNK inhibitor and J5+D10 groups showed significantly decreased expressions of p-NF-κB p105, p-NF-κB p65, and p-IκB compared to the DMSO group (Figure 3a-d). These results suggest that daphnetin and JNK inhibitors could regulate the activation of the NF-κB signaling pathway by inhibiting the phosphorylation of NF-κB p105, NF-κB p65, and IκB. JNK inhibitors enhance the inhibitory effect of daphnetin.

A549 cells were pretreated with JNK inhibitor for 1 h, then treated with DMSO and daphnetin with different concentrations for 24 h, and the cell proteins of Control group (0g/mL daphnetin), DMSO solvent control group and experimental group (10g/mL daphnetin, 5μmol/L JNK inhibitor, 5μmol/L JNK inhibitor+10g/mL daphnetin) were extracted, respectively. (a-d) The NF-κB signaling pathway-related molecules p-NF-κB p105, NF-κB p105, p-NF-κB p65, NF-κB p65, p-IκB, IκB were detected by Western Blot. * indicates the significant difference compared with DMSO group, #indicates the significant difference compared with D10 group, & indicates the significant difference compared with J5 group, and n.s indicates that there is no significant difference between the two groups (*p < 0.05, **p < 0.01, # *p* <0.05, & *p* <0.05).

### Daphnetin inhibits the proliferation of A549 cells

**Figure 4.**
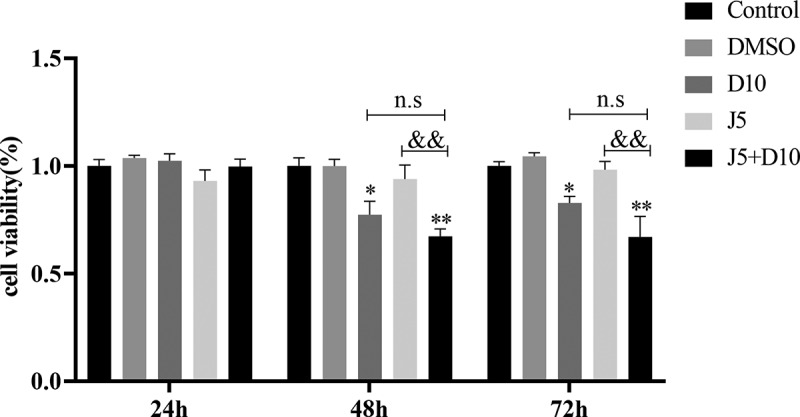
Effects of daphnetin and JNK inhibitor on proliferation of A549 cells.

The effects of daphnetin and JNK inhibitors on the proliferation of A549 cells were detected by adding DMSO (solvent control of daphnetin), JNK inhibitor and daphnetin to A549 cells for 24, 48, and 72 h, and the CCK8 reagent was added at different time points. The results showed that daphnetin had no effect on the proliferation of A549 cells in the experimental group when administered for 24 h, while daphnetin treated A549 cells for 48 h and 72 h, the proliferation of A549 cells (Figure 4). These results suggest that daphnetin inhibited the proliferation of A549 cells at 48 h and 72 h.

A549 cells were pretreated with JNK inhibitor for 1 h, then treated with DMSO and daphnetin with different concentrations for 24, 48, and 72 h. The effect of daphnetin and JNK inhibitor on proliferation of A549 cells was detected by CCK8 assay. * indicates the significant difference compared with DMSO group, & indicates the significant difference compared with J5 group, and n.s indicates that there is no significant difference between the two groups (*p < 0.05, **p < 0.01, &*p* <0.05, &&*p* <0.01).

### Daphnetin inhibits the invasion of A549 cells

**Figure 5.**
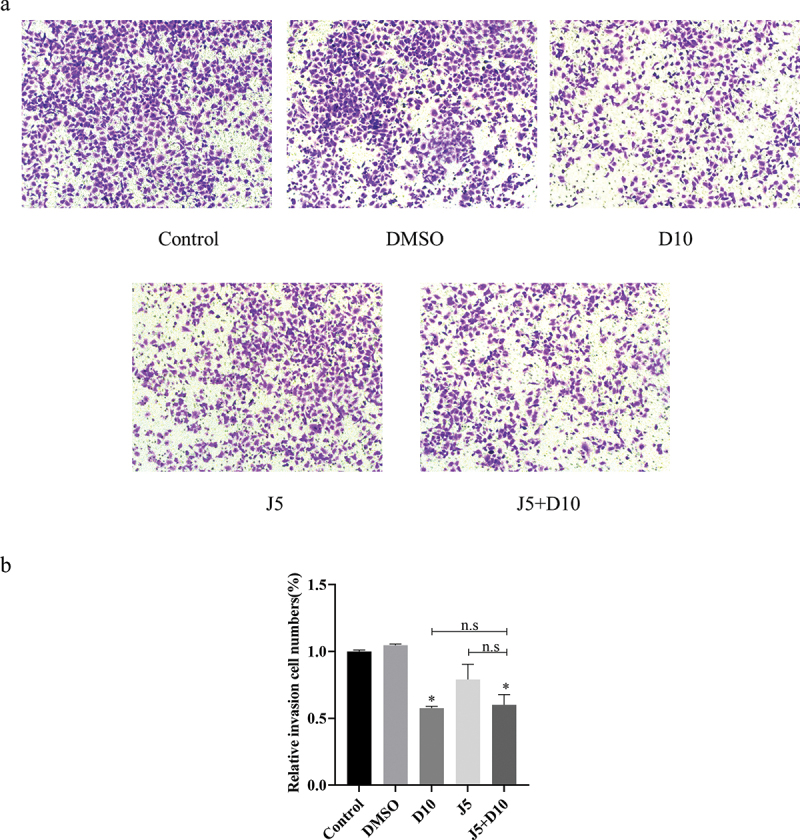
Effects of daphnetin and JNK inhibitor on invasion of A549 cells.

In this study, the transwell assay was used to investigate the effect of daphnetin on the invasive ability of lung adenocarcinoma A549 cells. The results showed that compared with the DMSO group, the daphnetin group invaded fewer cells into the ventricle, and the difference was statistically significant. Compared to the DMSO group, there was no significant difference in the number of cells invaded by the JNK inhibitor group (Figure 5a,b). These results indicate that daphnetin inhibited the invasion of lung adenocarcinoma A549 cells.

A549 cells were pretreated with JNK inhibitor for 1 h, then treated with DMSO and daphnetin with different concentrations. The effect of daphnetin and JNK inhibitors on invasion of A549 cells was detected by Transwell assay. * indicates the significant difference compared with DMSO group, and n.s indicates that there is no significant difference between the two groups (*p < 0.05, **p < 0.01).

### Daphnetin inhibits the migration of A549 cells

**Figure 6.**
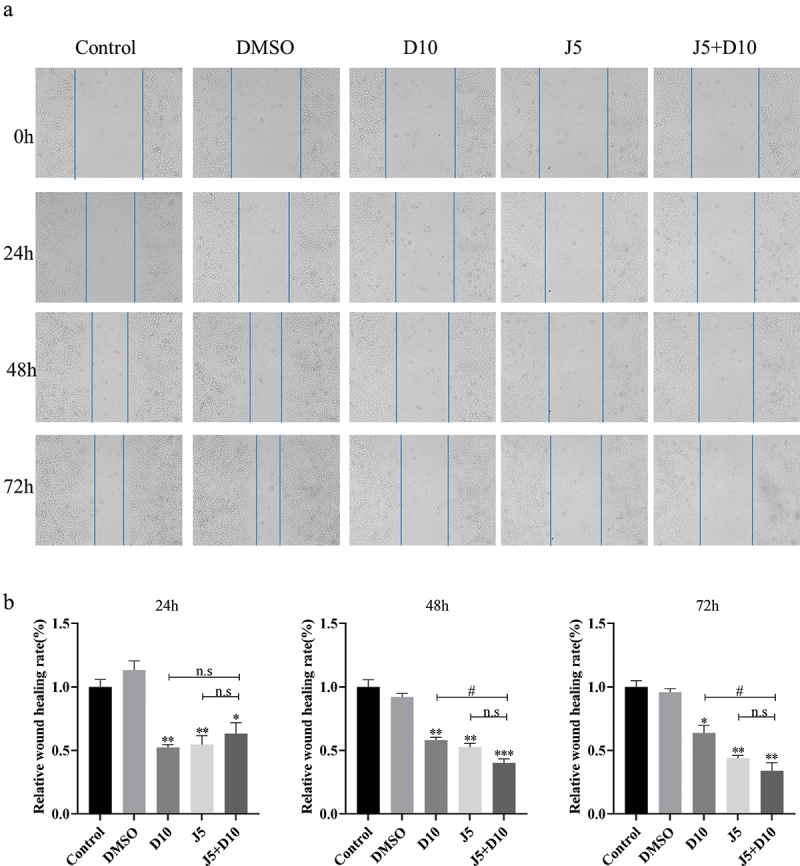
Effects of daphnetin and JNK inhibitor on migration of A549 cells.

Cancer cell migration and invasion are the early stages of cancer metastasis [13]. Scratch tests are commonly used to assess cell migration [14]. The results showed that compared with the DMSO group, the daphnetin and JNK inhibitor groups inhibited the reduction of scratch area at 24, 48, and 72 h, and the J5+D10 group also significantly inhibited the reduction of scratch area (Figure 6a,b). These results suggest that daphnetin inhibited the migration of lung adenocarcinoma A549 cells, which could be inhibited by the JNK pathway.

A549 cells were pretreated with JNK inhibitor for 1 h, then treated with DMSO and daphnetin with different concentrations for 24, 48, and 72 h. The effect of daphnetin and JNK inhibitor on migration of A549 cells was detected by wound healing assay. * indicates the significant difference compared with DMSO group, # indicates the significant difference compared with D10 group, and n.s indicates that there is no significant difference between the two groups (* *p* < 0.05, **p <0.01, #*p* <0.05).

### Daphnetin inhibits the colony formation of A549 cells

**Figure 7.**
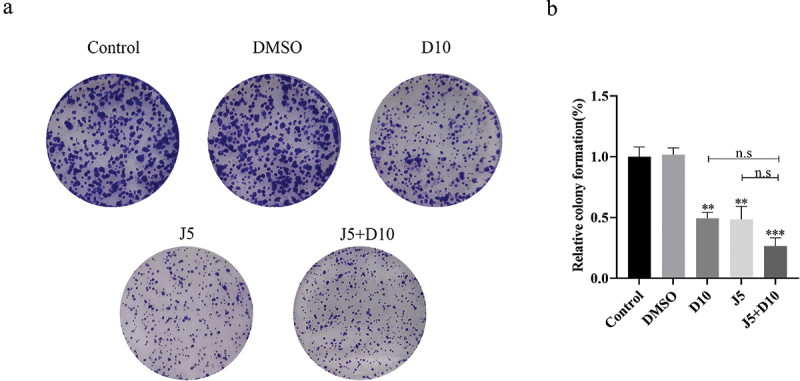
Effects of daphnetin and JNK inhibitor on colony formation of A549 cells.

The effect of daphnetin on the cloning ability of lung adenocarcinoma A549 cells was evaluated using a plate colony formation assay. Compared with the DMSO group, the colony formation ability in the daphnetin, JNK inhibitor, and J5+D10 groups was significantly reduced (Figure 7a,b). These results suggested that daphnetin could effectively inhibit colony formation in lung adenocarcinoma A549 cells, which may be involved in the regulation of the JNK pathway.

A549 cells were pretreated with JNK inhibitor for 1 h, then treated with DMSO and daphnetin with different concentrations. The effect of daphnetin and JNK inhibitor on colony formation of A549 cells was detected by colony formation experiment. *indicates the significant difference compared with DMSO group, and n.s indicates that there is no significant difference between the two groups (*p < 0.05, **p < 0.01).

## Discussion

Daphnetin has shown good anti-cancer properties in the treatment of cancers such as colon cancer, liver cancer, ovarian cancer and kidney cancer [2]. However, there are few studies on the treatment of lung cancer with daphnetin. In this study, lung adenocarcinoma A549 cells were selected as the research object to explore the anti-tumor effect of daphnetin on lung adenocarcinoma A549 cells. Cell signal transduction pathways are complex and variable, and the activation of relevant pathways can produce different biological effects, such as promoting tumor growth or inhibiting tumor growth [15]. It has been discovered that daphnetin, when used to treat various malignancies, blocks melanogenesis in B16F10 melanoma cells caused by α-melanocyte stimulating hormone (α-MSH) by inhibiting the PKA and ERK signaling pathways [16]. Therefore, we detected changes in the protein expression levels of JNK, p-JNK, ERK1/2, p-ERK1/2, p38 and p-p38 in lung adenocarcinoma A549 cells treated with daphnetin for 24 h. In this study, we found that daphnetin reduced the expression levels of p-JNK, p-ERK1/2 and p-p38 proteins, but did not change their total protein levels. These results suggested that daphnetin inhibited the activation of the MAPK pathway in lung adenocarcinoma A549 cells. In the study, it was found that after daphnetin was used, the expression of STAT3 protein in A549 cells did not change, but their expression of phosphorylated protein decreased obviously, so our research results showed that daphnetin could participate in regulating STAT3 pathway in A549 cells of lung adenocarcinoma. To further explore the potential relationship between the MAPK and NF-κB pathways, we used a JNK inhibitor for subsequent experiments. The results showed that daphnetin and a JNK inhibitor could regulate the STAT3 pathway by inhibiting the phosphorylation expression of STAT3. In this study, changes in NF-κBp105, IκB, NF-κBp65, and corresponding phosphorylated proteins were detected to explore the effect of daphnetin on the NF-κB pathway in A549 cells. Our results showed that daphnetin reduced the expression levels of p-NF-κB p105, p-IκB, and p-NF-κB p65. This was consistent with the results reported by Wang et al. JNK inhibitors could regulate the NF-κB pathway by decreasing the expression of p-NF-κB p105, p-IκB, and p-NF-κB p65. Both daphnetin and JNK inhibitors are involved in the regulation of the NF-κB pathway, and the JNK inhibitor enhances the inhibitory effect of daphnetin. The relationship between the STAT3 and NF-KB pathways in A549 cells affected by daphnetin requires further study. Animal models are needed to further investigate the anti-tumor effects of daphnetin.

Cell overproliferation is one of the most important signs of malignant tumors in humans. Uncontrolled growth and division of cells promotes cell proliferation, and an abnormal cell cycle leads to the occurrence of tumors [17]. Kimura et al. observed no inhibitory effect of daphnetin on the proliferation of osteosarcoma LM8 cells, but esculetin significantly inhibited the proliferation of LM8 cells for 12 and 24 h [18]. However, Jiménez-Orozco et others treated human breast cancer MCF-7 cells with daphnetin for 72 h before they showed inhibitory effects on cell proliferation [19]. Our results showed that daphnetin had no effect on cell proliferation after 24 h of treatment on A549 cells but inhibited proliferation after more than 48 h of treatment, and 5 µmol/L JNK inhibitor had no effect on cell proliferation. This is consistent with the literature. The results of the clone formation experiment showed that long-term daphnetin and JNK inhibitors could inhibit clone formation in A549 cells.

Metastasis is a hallmark of cancer and the cause of the largest number of cancer-related deaths [20]. The invasion and migration of cancer cells are an important step in cancer metastasis [13]. However, there are few studies on the effects of daphnetin on cell migration and invasion of lung adenocarcinoma A549 cells, and most of the previous reports have focused on the related mechanisms of the anti-proliferation and pro-apoptotic effects of daphnetin [10]. Therefore, our study examined the effects of daphnetin on cell migration and invasion of lung adenocarcinoma A549 cells using cell scratch and transwell invasion assay. The results suggested that daphnetin inhibited the migration and invasion of lung adenocarcinoma A549 cells to affect tumor progression, while the JNK inhibitor could inhibit cell migration by inhibiting the reduction of the scratch area.

## Conclusions

**Figure 8.**
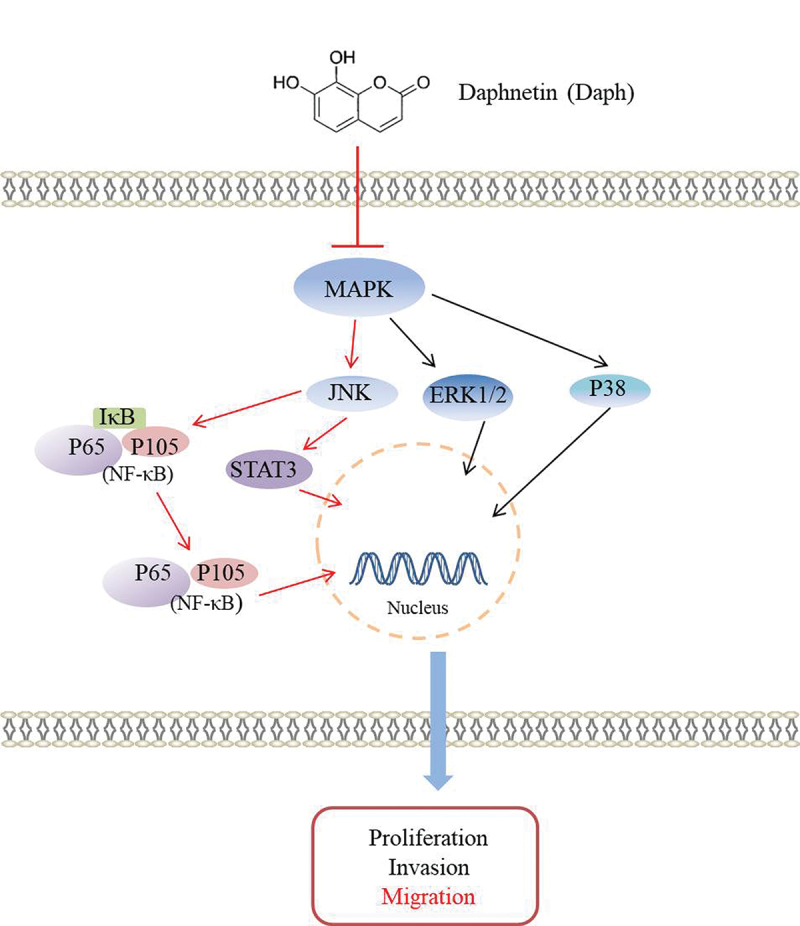
Daphnetin inhibits the MAPK/STAT3/NF-κB pathway mediated by A549 cell proliferation, migration and invasion signaling pathway.

In summary, the study on lung adenocarcinoma A549 cells suggested that daphnetin had anti-tumor effects. Daphnetin inhibits the proliferation, clonal formation, migration, and invasion of lung adenocarcinoma A549 cells by inhibiting the activation of the MAPK, NF-κB, and STAT3 signaling pathways. The JNK pathway is a key node in the regulation of STAT3 and NF-κB pathways by daphnetin. As shown in Figure 8, daphnetin inhibited the migration and clonal formation of A549 cells, mainly through the JNK pathway. These results provide a new research direction and theoretical basis for the use of daphnetin in the inhibition of lung adenocarcinoma.

## Data Availability

Data is available on request from the author, Rong Li.

## Abbreviations

MAPK: mitogen-activated protein kinase
NF-κB: nuclear factor κB
ERK1/2: extracellular signal-regulated kinase ½
JNK: c-Jun N-terminal kinase
Daph: daphnetin
STAT3: signal transducer and activator of transcription-3
AMPK: adenosine 5‘-monophosphate-activated protein kinase
